# Specific, simple and rapid detection of porcine circovirus type 2 using the loop-mediated isothermal amplification method

**DOI:** 10.1186/1743-422X-8-126

**Published:** 2011-03-18

**Authors:** Kai Zhao, Wei Shi, Fangting Han, Yan Xu, Lianlong Zhu, Yong Zou, Xiao Wu, Hong Zhu, Furong Tan, Shiru Tao, Xueming Tang

**Affiliations:** 1 Biotechnology Research Institute, Shanghai Academy of Agricultural Sciences, 2901 Beidi Road, Shanghai, 201106, People's Republic of China; 2 Institute of Animal Science and Veterinary Medicine, Shanghai Academy of Agricultural Sciences, 2901 Beidi Road, Shanghai, 201106, People's Republic of China; 3 Key Laboratory of Agricultural Genetics and Breeding, Shanghai Academy of Agricultural Sciences, 2901 Beidi Road, Shanghai, 201106, People's Republic of China; 4 College of Life and Environment Sciences, Shanghai Normal University,100 Guilin Road, Shanghai 200234, People's Republic of China

## Abstract

**Background:**

Porcine circovirus type 2 (PCV2) is the causative agent of postweaning multisystemic wasting syndrome (PMWS), and porcine dermatitis and nephropathy syndrome (PDNS). It has caused heavy losses in global agriculture in recent decades. Rapid detection of PCV2 is very important for the effective prophylaxis and treatment of PMWS.

**Results:**

A loop-mediated isothermal amplification (LAMP) assay was used to detect PCV2 in this study. Three pairs of primers were specially designed for recognizing eight distinct sequences of the ORF2 gene. This gene lies in the PCV2 virus genome sequence, and encodes the Rep protein that is involved in virus replication. Time and temperature conditions for amplification of PCV2 genes were optimized to be 55 min at 59°C. The analysis of clinical samples indicated that the LAMP method was highly sensitive. The detection limit for PCV2 by the LAMP assay was 10 copies, whereas the limit by conventional PCR was 1000 copies. The assay did not cross-react with PCV1, porcine reproductive and respiratory syndrome virus, porcine epidemic diarrhea virus, transmissible gastroenteritis of pigs virus or rotavirus. When 110 samples were tested using the established LAMP system, 95 were detected as positive.

**Conclusion:**

The newly developed LAMP detection method for PCV2 was more specific, sensitive, rapid and simple than before. It complements and extends previous methods for PCV2 detection and provides an alternative approach for detection of PCV2.

## Introduction

Porcine circovirus type 2 (PCV2) is a non-enveloped, circular, single-stranded DNA virus that belongs to the Circoviridiae family [1]. This virus is widespread in the commercial swine population, and is accepted as the causative agent of a number of diseases in these animals, such as postweaning multisystemic wasting syndrome (PMWS) [2], and porcine dermatitis and nephropathy syndrome (PDNS). These syndromes cause great losses to the pig industry. As a result, it is necessary to develop an effective method for detecting PCV2 to prevent these diseases. At present, many methods have been developed for the detection of this virus; among which, conventional PCR is commonly used [3]. However, the usefulness of PCR is limited by the presence of PCR inhibitors in the analysis of real biological samples. The wide range of inhibitors (including organic and inorganic substances such as detergents, antibiotics, phenolic compounds, enzymes, polysaccharides, fats, proteins and salts) reduces the amplification efficiency [4,5]. Apart from PCR, ELISA is one of the more common methods for virus detection [6]. It is hard to make a definitive diagnosis with ELISA in infected swine, because false-positive results may be included in the analysis [7]. In addition, real-time PCR is a good method to perform quantitative and qualitative analysis of PCV2 [8-11]. However, this method demands high-quality technical personnel. Hence, a rapid, sensitive and easy-to-operate method is still needed, especially for examination of PCV2.

Recently, a new technique called loop-mediated isothermal amplification (LAMP) has been developed, which can amplify nucleic acids with high specificity, sensitivity and rapidity under isothermal conditions [12]. The method is easily performed and highly specific for the target sequence because six independent sequences recognize the target sequence in the initial stage and four independent sequences amplify the target sequence in the later stage of the reaction [13,14]. LAMP assay has advantages in specificity, selectivity and rapidity over other nucleic acid amplification methods [13]. LAMP has been further advanced by using forward loop primers [15]. The method has been a valuable tool for the rapid diagnosis of infectious diseases in hospital laboratories and for the rapid detection of pathogenic microbes in food [16]. The use of LAMP for detecting PCV2 has been reported by Chen et al [17]. In their study, only four primers were used and no betaine was added to the LAMP assay. However, in our study, six primers containing two loop primers were used to amplify different regions of PCV2. It complemented and extended previous methods for PCV2 detection and provided an alternative approach for detection of PCV2. Therefore, the objectives of this study were to develop a LAMP assay for detecting ORF2 gene in PCV2 (which encodes Rep protein that is involved in virus replication), and to establish a more specific, sensitive, rapid and simple detection method for PCV2.

## Results

### Optimized temperature of LAMP assay for PCV2

**Figure 1:**
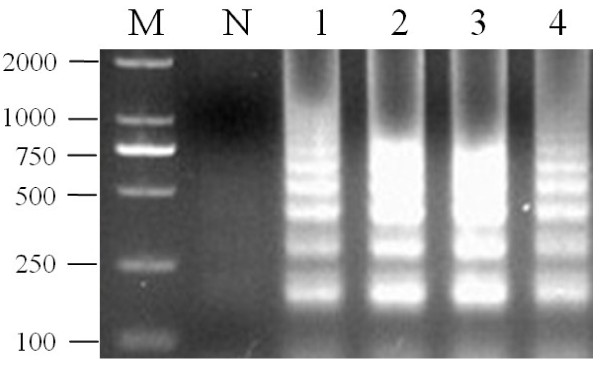
**Optimal temperature of the LAMP assay for detecting PCV2**. M: DL2000; lanes 1-4: LAMP carried out at 55, 57, 59 and 61°C, respectively. All the products were electrophoresed on 2% agarose gels and stained with Goldview.

On the basis of our temperature determination for the LAMP assay, the products amplified at 59°C exhibited slightly larger amounts of DNA than at other temperatures. Therefore, the optimal temperature of the LAMP reaction was 59°C. The Bst DNA polymerase was also work very well at this temperature (Figure 1). The optimal temperature for the PCR reaction was 54°C by incubating the reaction mixture.

### Specificity of the LAMP method

**Figure 2:**
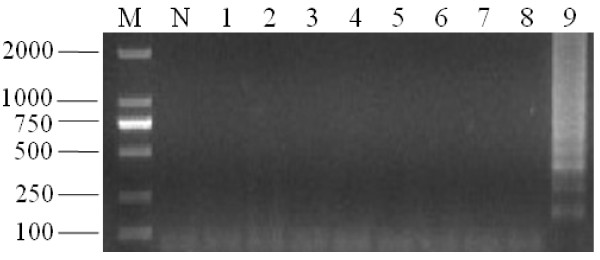
**Specificity of the LAMP reaction for detection of PCV2 gene**. **(A) **M, DL2000; lane 1, PCV1; lane 2, PCV1; lane 3, PPV; lane 4, PRV; lane 5, PEDV; lane 6, TEGV; lane 7, RV; lane 8, PRRSV; lane 9, PCV2; N, negative control. All the products were electrophoresed on 2% agarose gels and stained with Goldview.

**Figure 3:**
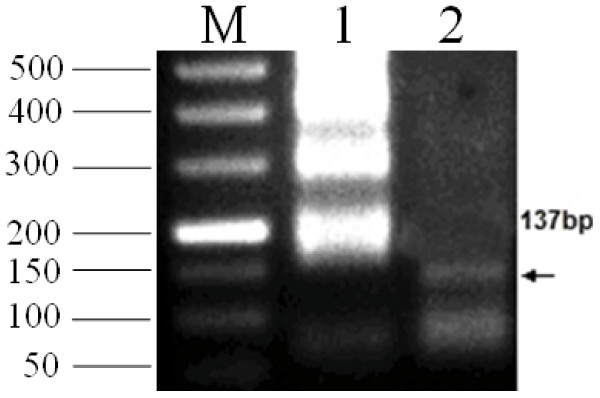
**Verification of the specific LAMP product of PCV2 by *Hae*III Digestion**. M, DL500; lane 1, LAMP product of PCV2; lane 2, specific LAMP product of PCV2 using *Hae*III digestion. All the products were electrophoresed on 2% agarose gels and stained with Goldview.

As expected, the typical ladder-like pattern products were only obtained in the assay using PCV2 genomic DNA samples as templates (Figure 2). In addition, the other virus species had no amplified products as well as the NTC (no template control). Figure 2 shows that the primers only amplified PCV2 strains and not other viruses. The sequencing results indicated that the amplified product length of two stains of PCV2 was 223 bp. The sequences showed 100% homology with the ORF2 gene of PCV2. The specificity of the LMAP product was confirmed by digestion of *Hae*III. A predictable product of the 137-bp motif was resolved on the agarose gel as expected (Figure 3, lane 2).

### Sensitivity of LAMP method and PCR assay for PCV2

**Figure 4:**
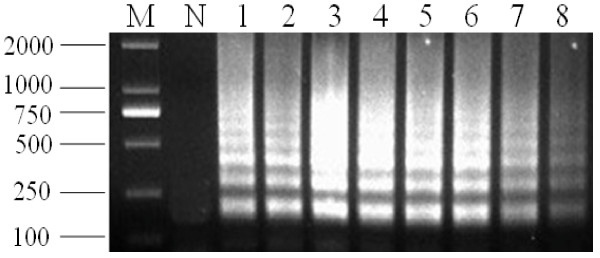
**Sensitivity of LAMP for detection of PCV2**. M, marker DL2000; N, negative control; lanes 1-8, LAMP carried out using the dilution series of PCV2 DNA, 10^8^, 10^7^, 10^6^, 10^5^, 10^4^, 10^3^, 100, and 10 copies, respectively. All the products were electrophoresed on 2% agarose gels and stained with Goldview.

**Figure 5:**
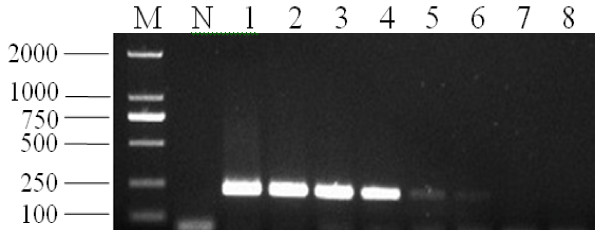
**Sensitivity of PCR for detection of PCV2**. M, marker DL2000; N, negative control; lanes 1-8, LAMP carried out using the dilution series of PCV2 DNA, 10^8^, 10^7^, 10^6^, 10^5^, 10^4^, 10^3^, 10^2 ^and 10 copies, respectively. All the products were electrophoresed on 2% agarose gels and stained with Goldview.

Comparative analysis of the sensitivity of PCV2 detection by the LAMP method and PCR was carried out using a dilution series of PCV2 DNA templates. The detection limit of the LAMP assay was 10 copies, which corresponded to 10 copies of DNA templates per reaction tube. The detection limit for the PCR was 1000 copies, which corresponded to 1000 copies of DNA templates per reaction tube. This suggested that the LAMP assay was 100-fold more sensitive than the conventional PCR (Figure 4 and Figure 5).

### PCV2 detection in clinical samples

One hundred and ten serum samples were analyzed using the LAMP method to determine whether they were infected by PCV2. PCV2 was detected in 95 clinical samples.

## Discussion

In this study, we developed a visual and rapid detection method for PCV2 using the optimized LAMP technique. Compared to conventional PCR analysis, the LAMP method has advantages such as time-saving, low cost and ease of operation.

The mature LAMP method was specific for PCV2 and had no amplified products for other viruses. We suggest that the set of primers had high specificity for the ORF2 gene of PCV2, according to PCR and other nucleic acid sequence-based methods. The LAMP method had high specificity for the target gene because six independent sequences (F1c, F2, F3, B1c, B2 and B3) recognized the target sequence in the initial stage, and four independent sequences (F1c, F2, B1c and B2) have been shown previously to amplify the target sequence in the later stage of the LAMP reaction [14]. However, the PCR assay only had one pair of primers to amplify the target gene. The big difference between the previous study and our research is that two loop primers take part in the LAMP reaction. The ORF2 gene of PCV2 has high specificity for detection of this virus.

The LAMP reaction can be performed under isothermal conditions without PCR thermal cyclers within a short time. In our study, the time for the whole LAMP reaction was <1 h. Moreover, the LAMP reaction did not require complicated pretreatment and expensive instrumentation. The reaction was more rapid than ELISA and real-time PCR. For that reason, the LAMP assay should be easy to use and widely applicable.

The LAMP assay developed had a detection limit of 10 copies for PCV2, which was 100-fold more sensitive than conventional PCR. The sensitivity of the LAMP assay was consistent with the reports of other virus species. For example, swine transmissible gastroenteritis coronavirus, H5 avian influenza virus, and yellow head virus [18-20].

When the LAMP assay was initiated, some measures should be taken to avoid false-negative results. Although the LAMP assay was an effective method for detection of PCV2 in clinical samples, laboratory contamination is one of the most serious problems in the LAMP reaction.

For detection of clinical samples, the procedure of DNA extraction was omitted. Serum samples were directly used as templates in the LAMP reaction. The sensitivity of the LAMP assay was less affected by the various components of the clinical samples than was the PCR. This reduces the time and cost of the test and simplifies many troublesome procedures in the LAMP assay. Most important, the superior tolerance to various compounds of the LAMP method makes it possible to popularize it in small hospitals, laboratories, private clinics and thremmatology.

Rapid detection of PCV2 by LAMP has been reported previously by Chen et al [18]. Their amplification method was finished in 60 min under isothermal conditions at 64°C by using a set of four primers that target the cap gene of PCV2. In our study, two more primers termed loop primers were added, which accelerated the LAMP reaction. Betaine was added to our LAMP reaction system to increase the melting speed. Moreover, porcine sera could be used as templates directly in the LAMP assay without DNA extraction, because the tolerance of the LAMP assay for biological substances was superior to other detection methods. Therefore, the DNA extraction step can be omitted from the LAMP assay [21].

## Conclusion

Our newly developed LAMP detection method for PCV2 is more specific, sensitive, rapid and simple than before. It complements and extends previous methods for PCV2 detection and provides an alternative approach for detection of PCV2.

## Material and methods

### Viral strains and clinical samples

Some viral strains were obtained from the Institute of Animal Science and Veterinary Medicine, Shanghai Academy of Agricultural Sciences. These viral strains contained PCV1, porcine parvovirus (PPV), pseudorabies virus (PRV), porcine epidemic diarrhea virus (PEDV), transmissible gastroenteritis of pigs virus (TGEV), rotavirus (RV), or porcine reproductive and respiratory syndrome virus (PRRSV). All the viral strains were used to conduct the LAMP assay. Swine sera were collected from an abattoir in Shanghai, and used as clinical samples for detection of PCV2 using the LAMP method.

### DNA and RNA extraction

DNA was extracted from a pure culture of cells infected by PCV2, according to the instructions of the Blood Viral DNA/RNA kit (BIOMIGA Inc, San Diego, CA, USA). The DNA template was used for optimizing the optimum temperature. Extraction of RNA from PEDV, TGEV, RV and PRRSV was conducted according to the instructions of the same kit. DNA extracted from PCV1, PPV and PRV and cDNA obtained by reverse transcription (Takara Corp., Japan) from RNA of PEDV, TGEV, RV and PRRSV were used to detect the specificicty of LAMP method for PCV2.

Decimal serial dilutions of PCV2 DNA were carried out in sterile double distilled water. Finally, the concentration of these DNA templates was from 10^8 ^to 10 copies. These different concentrations of DNA were used to determine the detection limit of PCV2 DNA.

### Primer design

Based on the ORF2 gene (GenBank accession no. EU921257.1) of PCV2 (strain BJ0804), six primers were designed using PrimerExplorer 3 (Table 1). The F3 and B3 primers were also used in the PCR reaction. The length of the amplified product was 223 bp. Contrary to previous methods, the newly added loop primers (LF and LB) significantly accelerated the LAMP reaction.

**Table 1:** Primers of LAMP assay for ORF2 of PCV2

Primer	Sequence
**F3**	**5'-**CACTTCGTAATGGTTTTTATTATTTA-**3'**
**B3**	**5'-**TCCACTATTGATTACTTCCAAC-**3'**
**FIP**	**5'-**CAGGAATACAATATCCGTGTAACCATTTTGGTTAAGTGGGGGGTCTT-**3'**
**BIP**	**5'-**GAGGCCTACGTGGTCTACATTTTTCAAACAACAAAAGAAATCAGCTATG-**3'**
**LF**	**5'-**AACCATGTATGTACAATTCAGAGAATTTAATC-**3'**
**LB**	**5'-**TTCCAGCAGTTTGTAGTCTCAGC-**3'**

### Reaction protocol for LAMP and PCR

LAMP was performed in a total 25-μL reaction mixture that contained 0.8 mM FIP and BIP, 0.2 mM of each of the outer primers (F3, forward outer primer; B3, backward outer primer), 0.4 mM LF and LB, 1.6 mM dNTPs, 0.8 M betaine (Sigma), 20 mM Tris-HCl (pH 8.8), 10 mM KCl, 10 mM (NH_4_)2SO_4_, 4 mM MgSO_4_, 0.1% Triton X-100, 8 U Bst DNA polymerase large fragment (New England Biolabs, Beverly, MA, USA). The LAMP reaction was carried out in a Jingda digital thermostatic water-bath (Jintan Jingda Apparatus Factory, China), as described previously [22-24]. PCR was carried out in a 25-μL reaction volume that contained 0.5 μM F3 and B3 and 0.75 mM dNTPs, 2.5 μM 10 × PCR buffer, 2.5 U Taq polymerase (Takara Corp.), and 1.0 μL extracted DNA or cDNA. After denaturation at 95°C for 5 min, the reaction system was subjected to thermal cycling in an Applied Biosystems 2720 thermal cycler (Foster City, CA, USA) for 35 cycles each of 95°C for 30 s, 55°C for 30 s and 72°C for 30 s, with a final elongation for 5 min at 72°C. LAMP and PCR amplified products were analyzed by electrophoresis on 2% (w/v) agarose gels and visualized by staining with Goldview, as described previously [17].

### Temperature determination of amplification

The LAMP reaction temperature (55-60°C) was optimized, and the assay was carried out for 45 min and terminated at 80°C for 3 min. The LAMP products were electrophoresed on a 2.0% agarose gel and visualized under UV light of Gel Doc XR+ ultraviolet light after Goldview staining, as described previously [25,26]. At the same time, the annealing temperature for PCR reaction was to be optimized.

### Specificity of the LAMP method

To establish the specificity of the LAMP detection method for PCV2, the DNA (PCV2, PCV1, PPV and PRV) and cDNA (PEDVD, TGEV, RV and PRRSV) were used as sample templates to initiate the reaction under the optimal conditions. Negative controls were included. The amplified products were digested with *Hae*III to confirm the reaction specificity, meanwhile, the headmost band of the products was sequenced by Shanghai Bioengineering Co. Ltd.

### Detection limits of LAMP and PCR for PCV2

The LAMP assay was initiated using different concentrations of DNA templates from 10^8 ^to 10 copies, following the optimized conditions described previously. At the same time, different concentrations of DNA were also used as templates for conventional PCR under optimized conditions. These amplified products were analyzed by electrophoresis on 2% (w/v) agarose gels and visualized under Gel Doc XR+ ultraviolet light by staining with Goldview. Conventional PCR was carried out as described above.

### Detection of PCV2 in clinical samples

A total of 110 serum samples were examined for presence of PCV2. These clinical samples were collected randomly and it was not known whether they were infected with PCV2. The same clinical samples were used directly in the LAMP assay and PCR reaction without DNA exaction, following the optimized conditions described above.

## Abbreviations

LAMP: loop-mediated isothermal amplification; PCV1: Porcine circovirus type 1: PCV2: porcine circovirus type 2; PEDV: porcine epidemic diarrhea virus; PMWS: postweaning multisystemic wasting syndrome; PPV: porcine parvovirus; PRRSV: porcine reproductive and respiratory syndrome virus; PRV: pseudorabies virus. RV: rotavirus; TGEV: transmissible gastroenteritis of pigs virus.

## Competing interests

The authors declare that they have no competing interests.

## Authors' contributions

KZ, WS, FTH and XMT participated in the design and carried out the majority of the experiments in the study and drafted the manuscript. YX, LLZ, YZ, XW, HZ, FRT and SRT helped to carry out the experiments and draft the manuscript. All authors read and approved the final manuscript.
